# Value Frameworks for Vaccines: Which Dimensions Are Most Relevant?

**DOI:** 10.3390/vaccines8040628

**Published:** 2020-10-28

**Authors:** Jeroen Luyten, Roselinde Kessels, Corinne Vandermeulen, Philippe Beutels

**Affiliations:** 1 KU Leuven, Department of Public Health and Primary Care, Leuven Institute for Healthcare Policy, Kapucijnenvoer 35, PO 7001, 3000 Leuven, Belgium; 2 Department of Data Analytics and Digitalization, Maastricht University, P.O. Box 616, 6200 MD Maastricht, The Netherlands; r.kessels@maastrichtuniversity.nl; 3 Department of Economics, City Campus, University of Antwerp, Prinsstraat 13, 2000 Antwerp, Belgium; 4 KU Leuven, Department of Public Health and Primary Care, Leuven University Vaccinology Centre, Kapucijnenvoer 35, PO 7001, 3000 Leuven, Belgium; Corinne.vandermeulen@kuleuven.be; 5 Centre for Health Economics Research and Modelling Infectious Diseases, Vaccine and Infectious Disease Institute, University of Antwerp, Universiteitsplein 1, 2610 Wilrijk, Belgium; philippe.beutels@uantwerpen.be

**Keywords:** vaccine evaluation, health technology assessment, evaluation space, public preferences, public involvement

## Abstract

In addition to more narrow criteria such as safety, effectiveness and cost-effectiveness, vaccines can also be evaluated based on broader criteria such as their economic impact, contribution to disease eradication objectives, caregiver aspects, financial protection offered, equity or social acceptability. We summarize a survey executed in a sample of the population (*n* = 1000) in Flanders, Belgium, in which we investigated support for using these broader criteria to evaluate vaccines for funding decisions. By means of both favourable and unfavourable framings of a hypothetical vaccine across 40 value dimensions, we find support for the view that people indeed consider a broad range of medical and socio-economic criteria relevant. Several of these are not incorporated in standard evaluation frameworks for vaccines. The different results we find for different framings highlight the importance of developing a consistent a priori value framework for vaccine evaluation, rather than evaluating vaccines on an ad hoc basis.

## 1. Introduction

Since the discovery of the first smallpox vaccine in the eighteenth century, public health policy makers now have at least fifty safe and effective vaccines at their disposal to prevent infectious diseases in their population [1]. However, as vaccine schedules cannot become overcrowded and budgets are finite, priority-setting between these vaccines is an inevitable reality of policy-making. To assist governments in making these difficult but inevitable choices, various decision-aid frameworks have been developed. These frameworks can be broadly categorized into two groups. On the one hand there are deliberative frameworks in which a structured set of general evaluation principles are defined for vaccines [2,3,4,5,6,7,8]. Examples of this deliberative approach were developed in 2014 by the WHO or in 2010 by the Health Council of the Netherlands. In the WHO framework, vaccines are evaluated based on the medical burden of the disease (its clinical aspects as well as its perception and alignment with national health policy goals), the performance, availability and economic impact of the vaccine and the impact of the vaccination program on the healthcare system (e.g., in terms of operational challenges) [3]. In the Dutch example a vaccine is evaluated on five dimensions: severity and size of the disease burden, effectiveness and safety of the vaccine, acceptability of the vaccine, efficiency of the vaccine and its priority relative to other vaccines [4]. The alternative is to adopt an algorithmic approach in which a scoring formula is used to estimate a vaccine’s value. The most elaborate representative of this approach is cost-utility analysis (CUA), expressing the value-for-money of a vaccine in a ratio of incremental costs per quality-adjusted life year (QALY) gained [9,10]. Criteria that are considered relevant in CUA are direct vaccine and disease treatment costs, indirect productivity losses and net health gains in QALYs due to vaccination, as well as the timing of the occurrence of the costs and effects. A concrete example of how a CUA-approach can produce rankings for vaccine priority setting can be found in the report “Vaccines for the 21st century” from the National Academy of Sciences (NAS) of the United States [11]. An updated and improved multiple criteria decision analysis (MCDA) framework from the NAS is “Smart Vaccines” [12,13].

Common to all these decision-aid frameworks for vaccine evaluation is the difficulty to define the right “evaluation space” for vaccines: the dimensions on which the performance of a vaccine should be evaluated. Vaccines have been shown to result in a broad range of potential consequences, beyond their rather narrow, clinically defined effects or their short-term healthcare costs and impact on labour productivity [14,15,16,17,18]. The COVID-19 crisis highlighted this fact and showed how infectious diseases can have enormous broader social, economic, political, psychological and ethical consequences. Before the pandemic, substantial literature had emerged highlighting how these broader consequences of infectious diseases are neglected in existing vaccine evaluation frameworks (see e.g., [17,19,20,21,22,23,24,25]).

At the same time it seems reasonable, in the first place for pragmatic reasons of a lack of reliable data or science, that not all consequences of vaccination can be included in vaccine evaluation (e.g., through health technology assessment). Certain criteria may be considered irrelevant for more fundamental reasons than pragmatism; because they are judged as irrelevant to the decision context. For instance, vaccines can be produced domestically by local vaccine manufacturers and funding a new vaccine might therefore affect employment and economic development. Anti-vaccination lobbies can fight the introduction of a particular vaccine and this might affect the broader public support for other vaccines as well [26]. Such consequences might be considered less relevant (or irrelevant) as a foundation to decide whether the vaccine should receive funding priority or not. Therefore, a good value framework for vaccines must find a balanced set of evaluation criteria that are “individually necessary and jointly sufficient” to assess the benefits offered relative to their costs.

To contribute to our understanding of which criteria are essential to be included in such a vaccine value framework, we set up a survey to investigate which criteria the general public considers most relevant to be used in vaccine funding decisions. Although, of course, lay people’s opinions are no substitute for the more technical judgment of experts, they can be an insightful “common sense” complement to it [27]. Moreover, it is important that decisions about which vaccine deserves funding are (at least partly) in line with public preferences. An unsupported vaccine evaluation framework that excludes important aspects or that includes (seemingly) less relevant aspects might increase mistrust in the decision-making process for vaccine funding, e.g., by creating perceptions propagated by anti-vaccination lobbies that governments are tied to pharmaceutical industries.

## 2. Materials and Methods

### 2.1. Survey

We constructed 40 criteria with potential relevance to vaccine evaluation, ranging from more obvious criteria such as safety or effectiveness to less obvious criteria such as contribution to disease eradication or impact on patient caregiving by family members. Of these criteria, 17 involved epidemiological and clinical aspects of the disease and the vaccine and 22 had a broader socio-economic nature. A respondent sample had to rate how relevant each criterion was to the decision whether a vaccine should be funded or not.

First, we familiarized respondents with the problem of budget scarcity and with the government’s challenge of setting vaccine priorities. Respondents received the following background: “The Flemish government has a fixed annual budget for vaccinations for the Flemish population. However, there are too many vaccines on the market and the budget is too small to pay for all possible vaccines. In other words, the government must choose and prioritize among the available vaccines. However, the question is: how should she do this? Based on which arguments?”

Next, we presented respondents with a fictitious vaccine that was candidate for public funding and invited them to assist the government in making a funding decision. More specifically, we asked respondents whether the vaccine’s performance on any of the 40 dimensions was relevant or not for the ultimate funding decision: “Imagine that the government is faced with the choice of either reimbursing a certain vaccine (called “VACCINEX”) or not reimbursing it. If she chooses VACCINEX, it means that there is less money available to pay for other vaccines. In the following, we will each time show you an argument that may be either important or unimportant for the funding decision”.

A simplistic example:

- If we tell you that VACCINEX is a safe or unsafe vaccine, that is probably a relevant argument.
- If we tell you that VACCINEX is in a nice or ugly package, that is probably not a relevant argument.

QUESTION: Should the government take this argument into account when deciding whether or not to reimburse VACCINEX? Or do you think this argument is irrelevant and should not play a role?

Respondents had to indicate the relevance to vaccine funding of each of the 40 value propositions on a Likert scale from 1 to 10. As the particular framing of the value proposition can affect people’s judgment [28], we decided to present criteria in two ways: through a favourable and an unfavourable framing. In the favourable version, the vaccine under evaluation scored well on the particular criterion. If this specific dimension is deemed relevant, then the vaccine would become more attractive for funding. In the unfavourable version, the vaccine scored poorly on the particular criterion. When the criterion is considered relevant, it becomes less attractive for funding. Both framings are important to understand the relevance of an overarching criterion. For example, the “vaccine safety” dimension could be deemed extremely important to guide decisions in case a vaccine induces a risk of serious side effects. But when a vaccine has no side effects at all, people might consider safety more self-evident and attribute lower importance to a vaccine’s safety profile. Table 1 shows all statements in their two formulations. We chose a “between subjects” design: each respondent evaluated either only 40 favourable or 40 unfavourable framings. All 40 statements were presented in random order to each respondent to avoid any order effects.

### 2.2. Sample

Via a market research agency (Research Now SSI–RN SSI) we collected two quasi-representative samples of 500 respondents, one for the favourable set of value propositions and one for the unfavourable one. Initially a stratified random sample of in total 1580 respondents was recruited from an online panel of 250,000 Flemish members (4% of the population), based on quotas for the Flemish population for the characteristics gender, age, educational level and geographical spread. Panel members received small incentives for participating in the research (e.g., vouchers). All these respondents completed our survey. The research agency then applied strict criteria and implemented tests to guarantee high data quality for our final sample. First, it excluded all “speedsters” (respondents who finish the survey in less than a third of the median time to complete the survey for the entire sample) and “straight-liners” (respondents who consistently give the same answer throughout the entire survey). Second, a test was added to the survey to be able to exclude those respondents who gave thoughtless answers. Two of the value propositions were repeated throughout the survey (so respondents completed 42 rating exercises in total). When there was more than 2 points difference between the scores given to both pairs of value propositions, respondents were automatically excluded from the final sample. For example, a score of 5/10 and 9/10 on one of the two repeated value propositions, and one of 5/10 and 8/10 on the other, led to automatic exclusion of the respondent. In total 580 respondents were excluded, leading to 1000 survey responses that were considered sufficiently valid and reliable for our analysis. Sample size calculation for comparing the means of two groups showed that a sample size of 500 respondents per group was more than sufficient to find significant differences of 0.5 in the mean respondent scores with a 95% power. Table 2 lists the main demographic characteristics of the included respondents.

## 3. Results

**Figure 1:**
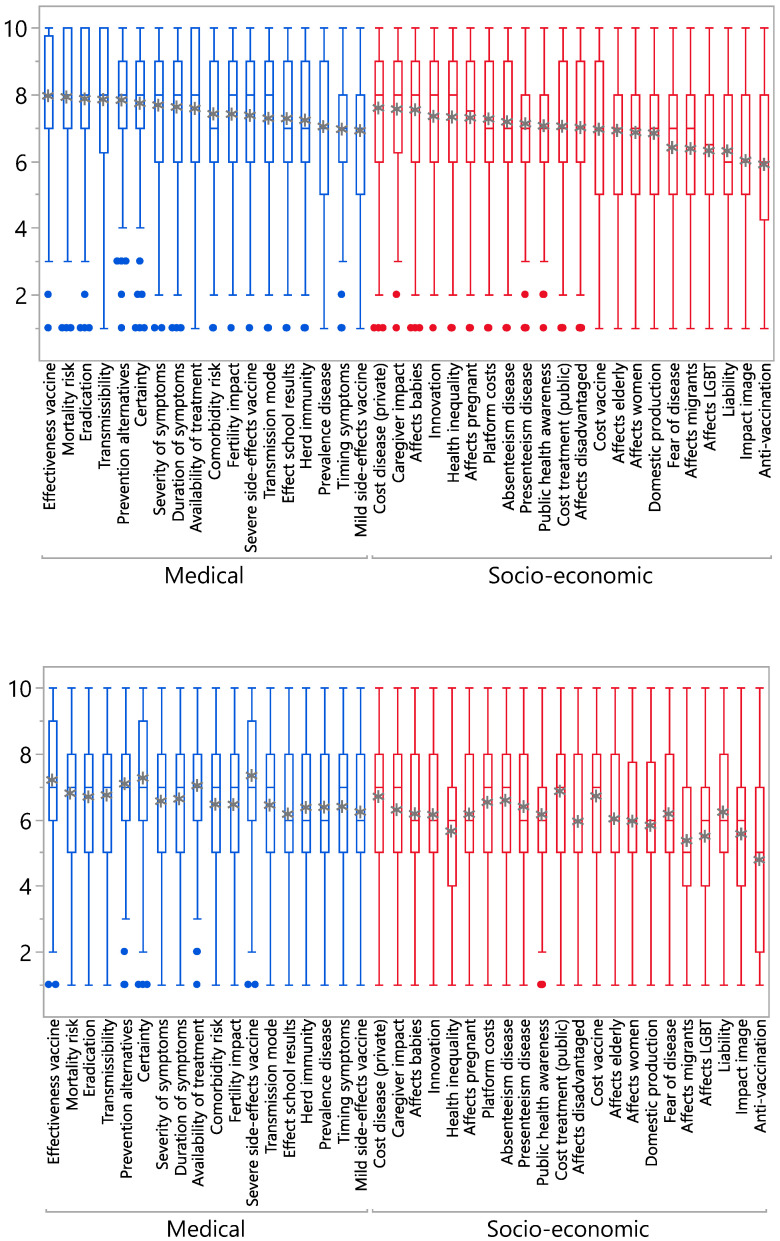
Boxplots with respondent scores on the favourable and unfavourable propositions. Upper panel: favourable propositions, lower panel: unfavourable propositions.

Figure 1 shows boxplots with the distributions of the respondent scores across the sample for the favourable and unfavourable statements. The asterisks on the boxplots denote the averages.

Three overall observations can be made. First, none of the 40 dimensions was dismissed as completely irrelevant by our sample, i.e., obtaining an average score below 5 in both positive and negative framings. The average of the positive items ranged from 5.9 (for the anti-vaccination proposition) to 7.9 (for the mortality proposition). The average of the negative items ranged from 4.8 (for the anti-vaccination proposition) to 7.3 (for the severe side-effects proposition). Only the negatively phrased anti-vaccination proposition had an average relevance score below 5. This indicates that our sample considers a broad range of criteria relevant to evaluate vaccines. Table 3 presents a ranking of the average scores of all statements in their positive and negative variants and connects them by means of letters. Statements with the same letter indicate that the average scores’ 95% confidence intervals overlap. This means that these average scores do not significantly differ from each other at the 5% level. The most relevant aspects were vaccine effectiveness, mortality risk, eradication potential, transmissibility, prevention alternatives, scientific certainty, severity of symptoms, duration of symptoms, cost disease (private), availability of treatment, caregiver impact, severe side-effects of the vaccine and whether the disease affects babies. The least relevant dimensions were whether the disease mainly affects migrants or LGBT members, legal liability for the state, impact on the country’s image and response by anti-vaccination groups.

Second, Table 3 shows that, overall, socio-economic aspects were considered less relevant than medical aspects related to (broader) safety and effectiveness. There were, however, a few notable exceptions. Whether the disease had high or low private and public treatment costs, whether there was a large impact on informal caregiving, a high vaccine cost and whether babies were particularly affected by the disease were all deemed of high relevance. Amongst the medical statements mild side-effects and timing of symptoms obtained lower scores.

Third, for all evaluation criteria, scores were markedly lower in the unfavourably phrased versions. This means that respondents judged a criterion as overall less relevant when the vaccine under evaluation was not scoring well on it. Vice versa, a criterion was judged as more relevant when the vaccine under evaluation obtained a favourable score on it. For instance, when vaccinex was considered to be protecting against a disease that is not very contagious, the criterion of transmissibility obtained an average score of 6.7. When the disease was contagious, the average score of transmissibility was 7.8.

We investigated whether there were groups of items for which the respondent scores were correlated. A factor analysis revealed two factors in each of the samples. Table 4 shows the factor loadings or correlation coefficients explaining how these factors load on, or correlate with the items’ scores. The first factor mainly describes the medical propositions and the second factor describes the socio-economic criteria. However, important exceptions to this classification were the socio-economic items about the private and public treatment cost of the disease, the caregiver impact of an infection, absenteeism, platform costs and the equity item whether the disease affects babies (or pregnant women). These socio-economic statements varied more alongside the factor of medical aspects. Likewise, the medical items of timing of symptoms and risk of mild side-effects of the vaccine varied more alongside the socio-economic factor. As can be seen in Table 4, the first factor obtained higher overall relevance scores than the second factor.

**Figure 2:**
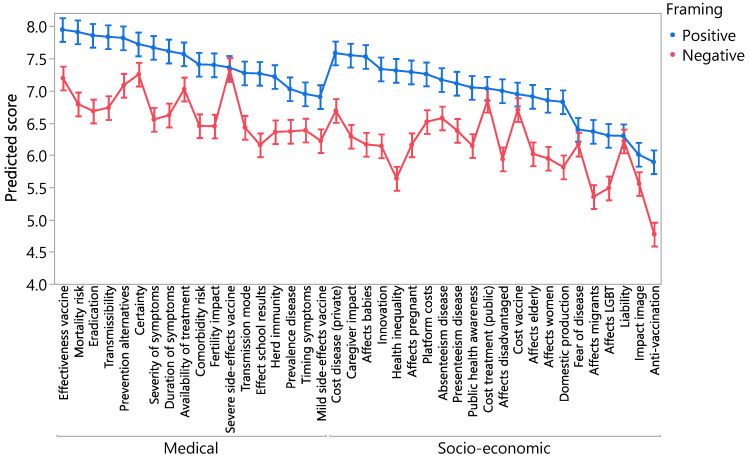
Predicted scores.

We constructed a general linear model to predict the respondent scores using item and framing (favourable and unfavourable) as categorical explanatory variables including the interaction between them. With an R-square of 95%, the model has a very high prediction power, where the predicted scores are the average scores from the boxplots in Figure 1. Figure 2 presents the predicted scores and their 95% confidence intervals. We clearly observe the relatively high scores of some socio-economic variables, such as caregiver impact and impact on babies. We also notice the systematically lower scores of the negative variants, except for a few items where scores overlap: risk of severe side-effects of the vaccine, treatment cost for the health system, vaccine cost, fear of the disease and legal liability.

## 4. Discussion

An important research challenge is to develop adequate evaluation frameworks for vaccines that manage to incorporate the broad range of consequences they can sort. In this study we investigated public support for various of these broader criteria. This is important because vaccine funding decisions are impactful for population health and, as such, they can affect everyone, directly or indirectly. By recommending funding priorities, they determine which infectious disease risks we, as a society, consider a priority to prevent but also which diseases we choose to tolerate. To be “procedurally fair”, these frameworks must be based on “relevant” criteria only: priorities may neither be based on irrelevant information nor on partial, incomplete evaluation only. This is for instance acknowledged in the ethical “Accountability for Reasonableness” (A4R) framework for fair priority setting in healthcare [29].

Our sample did not limit itself to a narrow set of basic criteria (safety, effectiveness, cost-effectiveness) but instead considered the relevance of a broad range of salient and less salient aspects. All the studied criteria, except perhaps the anti-vaccination movement’s response, were considered important (average relevance score above 5/10). Certain socio-economic aspects were considered of particular relevance. For instance, a vaccine’s impact in terms of affecting caregiver burden, treatment cost for the disease, and whether newborns are the main target group played a similar role in regard to the impact of medical aspects such as duration, type or severity of disease symptoms. Amongst the medical aspects, our sample attributed a remarkably high importance to the disease eradication potential of a vaccine and the degree of scientific certainty around the vaccine’s effects.

How do these findings relate to available decision-aid frameworks? Whereas many of the criteria that we studied (disease burden in infected patients, infectivity, duration of symptoms, treatment options, but also the effectiveness of the vaccine and the risk of severe side-effects) are included in deliberative frameworks [5] as well as in CUA [10], several of the considerations that were deemed important are neglected in those evaluation frameworks. For instance, the impact on informal caregiving was considered an important criterion, but this is often given insufficient attention in vaccine evaluation [30]. The potential for disease eradication and the degree of scientific certainty regarding the effect of the vaccine were deemed relevant evaluation criteria. Specific guidelines exist for taking into account uncertainty [31], but they are often not fully followed. Broader aspects of health that can be affected by infectious diseases, such as cognition and fertility (both not included in QALYs [32]) were also found to be more or less relevant by a large proportion of the sample.

A second finding is the large difference that was found in the relevance of a criterion depending on whether the vaccine was presented as more or less favourable. The relevance score of a criterion for vaccine funding decisions was systematically considered lower when the vaccine was presented in a less favourable way. This finding highlights the importance of developing a consistent value framework for vaccines in which the weight of an exhaustive list of criteria is established a priori, rather than evaluating vaccines on an ad hoc basis where the weight or relevance of criteria is determined during the vaccine evaluation process itself. Our study suggests that without using pre-established value frameworks, there is a risk that the focus will be disproportionally on those characteristics that are considered to be most relevant by policy makers to the vaccine under evaluation. Other important criteria that are nonetheless important to other vaccines, which are competing for the same resources, can as such be neglected.

Our study had limitations. We investigated lay people’s opinion regarding a complicated subject. Answers are merely indicative of which criteria matter to the public in vaccine evaluation and are meant to be used in discussions about what an “optimal” value framework for vaccines looks like. We used an online panel for this study and we think this is sufficient for our purpose, but panel membership may be associated with particular characteristics that reduce representativeness (e.g., internet access). Although we did make many efforts to exclude respondents that did not provide thoughtful answers, we still cannot fully distinguish between better and worse informed respondents.

## 5. Conclusions

In conclusion, vaccines can sort a broad range of consequences but it is not always clear which of these must be incorporated in funding decisions. Our study suggests that people indeed consider many broader aspects of vaccines as relevant and this suggests that they support a broad value framework for vaccines. Our study also indicates that it is important to determine the elements of this framework beforehand. These insights can be valuable to researchers in the fields of health technology assessment, MCDA or evidence-based decision-making who aim to capture the value of vaccines in new evaluation frameworks.

## Figures and Tables

**Table 1:** Value propositions, favourably and unfavourably framed.

Criterion	Favourable Framing	Unfavourable Framing
**Medical aspects of disease and vaccine**		
Mortality risk	Anyone who becomes infected with the disease (against which VACCINEX protects) has a high risk of dying.	Those who become infected with the disease (against which VACCINEX protects) do not run a risk of dying.
Severity of symptoms	Those who become infected with the disease (against which VACCINEX protects) get severe symptoms.	Those who become infected with the disease (against which VACCINEX protects) will only have mild symptoms.
Duration of symptoms	The symptoms of the disease (against which VACCINEX protects) persist for life.	The symptoms of the disease (against which VACCINEX protects) are temporary.
Comorbidity risk	Those who get the disease (against which VACCINEX protects) are later also more likely to get other diseases.	Those who get the disease (against which VACCINEX protects) do not run a higher risk of developing other diseases later on.
Transmis-sibility	The virus (against which VACCINEX protects) is very contagious and it will infect many people in Flanders.	The virus (against which VACCINEX protects) is not contagious and will infect few people in Flanders.
Timing symptoms	Anyone who becomes infected with the disease (against which VACCINEX protects) immediately develops symptoms. In other diseases, these only break through after a few years.	Those who become infected with the disease (against which VACCINEX protects) will only develop symptoms after a few years. In other diseases, these often break through immediately.
Eradication potential	If many people are vaccinated with VACCINEX, the disease can be eradicated so that future generations no longer need to be vaccinated.	No matter how many people are vaccinated with VACCINEX, the disease will never be eradicated. Future generations will also have to be vaccinated.
Availability of treatment	Those who become infected with the disease (against which VACCINEX protects) cannot be treated for this.	Those who become infected with the disease (against which VACCINEX protects) can be treated for this.
Prevention alternatives	Vaccination with VACCINEX is the only effective way to prevent the disease.	The disease against which VACCINEX protects can also be avoided in other ways than through vaccination.
Prevalence disease	The disease (against which VACCINEX protects) is common in Flanders.	The disease (against which VACCINEX protects) is rare in Flanders.
Transmis-sion mode	The disease (against which VACCINEX protects) is not spread through sexual contact but through the respiratory tract.	The disease (against which VACCINEX protects) is not spread through the respiratory tract, but through sexual contact.
Fertility impact	Anyone who becomes infected with the disease (against which VACCINEX protects) suffers damage to fertility and can therefore have an effect on both the existence and health of the offspring.	Anyone who is infected with the disease (against which VACCINEX protects) does not suffer any damage to fertility and this will therefore have no effect on the existence or health of the offspring.
Effectiveness vaccine	VACCINEX offers highly effective disease protection.	VACCINEX offers little effective disease protection.
Scientific certainty	There is good scientific certainty about the effects of VACCINEX.	There is much scientific uncertainty about the effects of VACCINEX.
Mild side-effects	Anyone who is vaccinated with VACCINEX will not experience any mild side-effects (e.g., headache, mild fever).	Anyone who is vaccinated with VACCINEX is at risk of some mild side-effects (e.g., headache, mild fever).
Severe side-effects	Anyone who is vaccinated with VACCINEX will not experience any serious side-effects (e.g., convulsions, severe allergic reaction).	Anyone who is vaccinated with VACCINEX is at risk of some serious side-effects (e.g., convulsions, severe allergic reaction).
Herd immunity	When many people are vaccinated with VACCINEX, “herd immunity” occurs and unvaccinated people are indirectly protected against the disease (because the risk of infection decreases).	No matter how many people are vaccinated with VACCINEX, no “herd immunity” occurs. Consequently, only the vaccinated themselves are protected. There is no indirect protective effect for unvaccinated persons.
**Socio-economic aspects of disease and vaccine**		
Cost vaccine	VACCINEX is inexpensive compared to other vaccines.	VACCINEX is expensive compared to other vaccines.
Cost disease (public)	Anyone who becomes infected with the disease (against which VACCINEX protects) must undergo expensive treatment and this costs the government a lot of money.	Those who become infected with the disease (against which VACCINEX protects) must undergo inexpensive treatment. This does not cost the government much money.
Cost disease (private)	Anyone who becomes infected with the disease (against which VACCINEX protects) must undergo treatment and this will cost the patient a lot of money.	Anyone who becomes infected with the disease (against which VACCINEX protects) must undergo treatment, but this will cost the patient little money.
Platform costs	VACCINEX is easy and inexpensive to implement because it can be linked to other vaccinations already rolled out. As a result, no separate doctor’s appointment has to be made.	VACCINEX is difficult and more expensive to implement because it cannot be linked to other vaccinations that have already been rolled out. This means that a separate doctor’s appointment must always be made.
Productivity costs: absenteeism disease	Those who become infected with the disease (against which VACCINEX protects) cannot go to work for a long time. This lost time costs society a lot of money.	Those who become infected with the disease (against which VACCINEX protects) can quickly return to work. The lost time does not cost society a lot of money.
Productivity costs: presenteeism disease	Those who become infected with the disease (against which VACCINEX protects) are much less productive at work for a long time.	Those who become infected with the disease (against which VACCINEX protects) are equally productive at work.
Caregiver impact	Those who become infected with the disease (against which VACCINEX protects) need long-term help from their partner (informal care).	Those who become infected with the disease (against which VACCINEX protects) do not need help from their partner (no informal care).
Effect school results	If children become infected with the disease (against which VACCINEX protects), it has serious effects on their study results.	If children become infected with the disease (against which VACCINEX protects), this has no effect on their study results.
Domestic production	VACCINEX is produced by a Flemish company and therefore provides more employment in Flanders than vaccines produced abroad.	VACCINEX is produced by a foreign company and therefore provides less employment in Flanders than vaccines that are produced here.
Innovation stimulus	VACCINEX reimbursement provides a scientific stimulus and leads to more scientific innovation.	VACCINEX reimbursement does not provide scientific incentives and does not lead to more scientific innovation.
Goodwill and image	The disease (against which VACCINEX protects) is bad for the international image of Flanders and that has economic consequences (e.g., on tourism).	The disease (against which VACCINEX protects) does not damage the international image of Flanders and has no impact on e.g., tourism.
Health inequality impact	VACCINEX helps to reduce health inequalities between rich and poor.	VACCINEX does not help to reduce health inequalities between rich and poor.
Public health awareness	VACCINEX improves the general awareness of public health and the efforts it requires.	VACCINEX does not improve public awareness of public health and the efforts it requires.
Perception and fear	People have an excessive fear for the disease against which VACCINEX protects.	People do not have an excessive fear for the disease against which VACCINEX protects.
Anti-vaccination	If VACCINEX is refunded, there will be no protests from anti-vaccination groups.	If VACCINEX is refunded, protests will arise from anti-vaccination groups.
Legal liability	VACCINEX exposes the government to potential litigation less than other vaccines.	VACCINEX exposes the government to potential litigation more than other vaccines.
Target group: affects dis-advantaged	The disease (against which VACCINEX protects) is more common in disadvantaged groups.	The disease (against which VACCINEX protects) is rare in disadvantaged groups.
Target group: affects migrants	The disease (against which VACCINEX protects) is more common in migrants.	The disease (against which VACCINEX protects) is rare in migrants.
Target group: affects babies	The disease (against which VACCINEX protects) is more common in babies and young children.	The disease (against which VACCINEX protects) is rare in babies and young children.
Target group: affects elderly	The disease (against which VACCINEX protects) is more common in elderly people.	The disease (against which VACCINEX protects) is rare in elderly people.
Target group: affects LGBT	The disease (against which VACCINEX protects) is more common in people from the LGBT (lesbian-gay-bisexual-transgender) community.	The disease (against which VACCINEX protects) is rare in people from the LGBT (lesbian-gay-bisexual-transgender) community.
Target group: affects women	The disease (against which VACCINEX protects) is more common in women.	The disease (against which VACCINEX protects) is rare in women.
Target group: affects pregnant women	The disease (against which VACCINEX protects) is more common in pregnant women.	The disease (against which VACCINEX protects) is rare in pregnant women.

**Table 2:** Sample characteristics.

Characteristic		Sample 1		Sample 2	
		Favourable Framing		Unfavourable Framing	
		*n*	%	*n*	%
**Gender**	Male	245	49%	245	49%
	Female	255	51%	255	51%
**Age**	18–24	55	11%	54	11%
	25–34	75	15%	75	15%
	35–44	80	16%	80	16%
	45–54	95	19%	95	19%
	55–64	155	31%	159	32%
	65–75	40	8%	33	7%
**Educational Level**	None	8	2%	9	2%
	Primary school	45	9%	40	8%
	Secondary school	302	60%	306	61%
	Higher, non-university	111	22%	92	18%
	University or post-university	33	7%	46	9%
	Other	1	0%	7	1%
**Province**	Antwerp	140	28%	140	28%
	Limburg	65	13%	65	13%
	East-Flanders	115	23%	115	23%
	Flemish-Brabant	85	17%	85	17%
	West-Flanders	95	19%	95	19%
**Monthly Net Income**	< €1000	77	15%	68	14%
	€1000–€1200	55	11%	62	12%
	€1200–€1400	50	10%	61	12%
	€1400–€1600	47	9%	60	12%
	€1600–€1800	52	10%	44	9%
	€1800–€2000	54	11%	63	13%
	€2000–€2300	60	12%	49	10%
	€2300–€2900	47	9%	43	9%
	€2900–€3400	39	8%	14	3%
	€3400–€4000	9	2%	11	2%
	> €4000	10	2%	23	5%
**Civil State**	Single	160	32%	164	33%
	Factual cohabitant	59	12%	54	11%
	Married	176	35%	177	35%
	Widowed	10	2%	8	2%
	Legal cohabitant	42	8%	37	7%
	Divorced	53	11%	60	12%
**TOTAL**		**500**	**100%**	**500**	**100%**

**Table 3:** Analysis of variance (ANOVA) for analysing differences in the mean scores for the 40 statements.

A. Favourable Propositions	Mean																B. Unfavourable Propositions	Mean																	
Effectiveness vaccine	7.9	A															Severe side-effects vaccine	7.3	A																
Mortality risk	7.9	A															Scientific certainty	7.3	A	B															
Eradication	7.9	A	B														Effectiveness vaccine	7.2	A	B	C														
Transmissibility	7.8	A	B	C													Prevention alternatives	7.1	A	B	C	D													
Prevention alternatives	7.8	A	B	C													Availability of treatment	7.0	A	B	C	D	E												
Scientific certainty	7.7	A	B	C	D												Cost treatment (public)	6.9	A	B	C	D	E	F											
Severity of symptoms	7.7	A	B	C	D	E											Mortality risk	6.8		B	C	D	E	F	G										
Duration of symptoms	7.6	A	B	C	D	E	F										Transmissibility	6.7		B	C	D	E	F	G	H									
Cost disease (private)	7.6	A	B	C	D	E	F										Cost vaccine	6.7			C	D	E	F	G	H									
Availability of treatment	7.6	A	B	C	D	E	F										Cost disease (private)	6.7			C	D	E	F	G	H	I								
Caregiver impact	7.6	A	B	C	D	E	F	G									Eradication	6.7			C	D	E	F	G	H	I								
Target group: Affects babies	7.5	A	B	C	D	E	F	G	H								Duration of symptoms	6.6				D	E	F	G	H	I	J							
Comorbidity risk	7.4		B	C	D	E	F	G	H	I							Absenteeism disease	6.6				D	E	F	G	H	I	J							
Fertility impact	7.4		B	C	D	E	F	G	H	I	J						Severity of symptoms	6.6				D	E	F	G	H	I	J							
Severe side-effects vaccine	7.4		B	C	D	E	F	G	H	I	J						Platform costs	6.5					E	F	G	H	I	J	K						
Innovation	7.3			C	D	E	F	G	H	I	J	K					Comorbidity risks	6.5						F	G	H	I	J	K	L					
Health inequality	7.3				D	E	F	G	H	I	J	K	L				Fertility impact	6.4						F	G	H	I	J	K	L					
Target group: affects pregnant women	7.3				D	E	F	G	H	I	J	K	L				Transmission mode	6.4						F	G	H	I	J	K	L					
Transmission mode	7.3				D	E	F	G	H	I	J	K	L				Timing symptoms	6.4						F	G	H	I	J	K	L					
Effect school results	7.3				D	E	F	G	H	I	J	K	L				Presenteeism disease	6.4						F	G	H	I	J	K	L					
Platform costs	7.3				D	E	F	G	H	I	J	K	L				Prevalence disease	6.4						F	G	H	I	J	K	L					
Herd immunity	7.2					E	F	G	H	I	J	K	L				Herd immunity	6.4						F	G	H	I	J	K	L					
Absenteeism disease	7.2					E	F	G	H	I	J	K	L				Caregiver impact	6.3							G	H	I	J	K	L	M				
Presenteeism disease	7.1						F	G	H	I	J	K	L				Mild side-effects vaccine	6.2								H	I	J	K	L	M				
Public health awareness	7.1							G	H	I	J	K	L				Liability	6.2								H	I	J	K	L	M				
cost treatment (public)	7.0								H	I	J	K	L				Fear and perception	6.2									I	J	K	L	M	N			
Prevalence disease	7.0									I	J	K	L				Target group: affects babies	6.2									I	J	K	L	M	N			
Target group: affects disadvantaged	7.0									I	J	K	L				Target group: affects pregnant women	6.2									I	J	K	L	M	N			
Timing symptoms	7.0									I	J	K	L				Effect school results	6.2									I	J	K	L	M	N			
Cost vaccine	6.9									I	J	K	L				Public health awareness	6.1										J	K	L	M	N			
Target group: affects elderly	6.9									I	J	K	L				Innovation	6.1										J	K	L	M	N			
Mild side-effects vaccine	6.9										J	K	L				Target group: affects elderly	6.0											K	L	M	N	O		
Target group: affects women	6.9											K	L	M			Target group: affects women	5.9												L	M	N	O		
Domestic production	6.8												L	M			Target group: affects disadvantaged	5.9												L	M	N	O		
Fear and perception	6.4													M	N		Domestic production	5.8													M	N	O	P	
Target group: affects migrants	6.4													M	N	O	Health inequality	5.6														N	O	P	
Target group: affects LGBT	6.3														N	O	Impact image	5.6															O	P	
Legal liability	6.3														N	O	Target group: affects LGBT	5.5															O	P	
Impact image	6.0														N	O	Target group: affects migrants	5.4																P	
Anti-vaccination	5.9															O	Anti-vaccination	4.8																	Q

**Table 4:** Factor loadings from a factor analysis on the respondent scores for the favourable and unfavourable statements.

	Favourable Statements		Unfavourable Statements	
	Factor 1	Factor 2	Factor 1	Factor 2
**Medical aspects**				
Effectiveness vaccine	**0.81**	0.14	**0.82**	−0.02
Mortality risk	**0.87**	0.12	**0.69**	0.35
Eradication	**0.76**	0.21	0.42	0.48
Transmissibility	**0.83**	0.15	**0.69**	0.22
Prevention alternatives	**0.79**	0.14	**0.73**	0.14
Certainty	**0.75**	0.16	**0.72**	−0.02
Severity of symptoms	**0.78**	0.20	**0.61**	0.36
Duration of symptoms	**0.77**	0.22	**0.59**	0.35
Availability of treatment	**0.72**	0.11	**0.71**	0.26
Comorbidity risk	**0.72**	0.24	0.48	**0.53**
Fertility impact	**0.59**	0.42	0.46	0.49
Severe side-effects vaccine	**0.62**	0.31	**0.69**	0.03
Transmission mode	0.48	0.45	0.41	**0.51**
Effect school results	**0.53**	0.43	0.33	**0.59**
Herd immunity	**0.59**	0.29	0.45	**0.50**
Prevalence disease	0.43	0.44	**0.55**	0.41
Timing symptoms	0.42	**0.58**	0.43	**0.52**
Mild side-effects vaccine	0.38	0.48	0.38	**0.50**
**Socio-economic aspects**				
Cost disease (private)	**0.63**	0.30	**0.51**	0.46
Caregiver impact	**0.69**	0.32	0.43	**0.56**
Affects babies	**0.67**	0.33	0.40	**0.63**
Innovation	0.40	**0.51**	0.37	**0.50**
Health inequality	0.45	0.46	0.10	**0.64**
Affects pregnant	**0.53**	0.41	0.37	**0.66**
Platform costs	0.48	0.37	**0.50**	0.46
Absenteeism disease	0.45	0.39	**0.57**	0.34
Presenteeism disease	0.43	**0.55**	0.48	**0.50**
Public health awareness	0.37	**0.52**	0.35	**0.53**
Cost treatment (public)	0.42	0.27	**0.58**	0.27
Affects disadvantaged	0.33	**0.59**	0.26	**0.69**
Cost vaccine	0.43	0.36	**0.51**	0.34
Affects elderly	0.35	**0.58**	0.25	**0.72**
Affects women	0.40	**0.60**	0.35	**0.64**
Domestic production	0.19	**0.57**	0.20	**0.59**
Fear of disease	0.12	**0.49**	**0.50**	**0.51**
Affects migrants	0.14	**0.63**	0.04	**0.70**
Affects LGBT	0.17	**0.61**	0.09	**0.76**
Liability	0.11	**0.61**	0.40	0.41
Impact image	0.04	0.45	0.12	**0.62**
Anti-vaccination	−0.08	0.48	−0.09	**0.64**

Note: Important factor loadings larger than or equal to 50% are indicated in bold.
